# Treatments and Patient Outcomes Following Stroke Center Expansion

**DOI:** 10.1001/jamanetworkopen.2024.44683

**Published:** 2024-11-13

**Authors:** Yu-Chu Shen, Anthony S. Kim, Renee Y. Hsia

**Affiliations:** 1 Department of Defense Management, Naval Postgraduate School, Monterey, California; 2 National Bureau of Economic Research, Cambridge, Massachusetts; 3 UCSF Weill Institute of Neurosciences, Department of Neurology, University of California, San Francisco; 4 Department of Emergency Medicine, University of California, San Francisco; 5 Philip R. Lee Institute for Health Policy Studies, University of California, San Francisco

## Abstract

**Importance:**

It is unclear how certified stroke center expansion contributes to improved access to stroke treatment and patient outcomes, and whether these outcomes differ by baseline stroke center access.

**Objective:**

To examine changes in rates of admission to stroke centers, receipt of thrombolysis and mechanical thrombectomy, and mortality when a community gains a newly certified stroke center within a 30-minute drive.

**Design, Setting, and Participants:**

This cohort study compared changes in patient outcomes when a community (defined by area zip code) experienced a stroke center expansion relative to the same community type that did not experience a change in access. Medicare fee-for-service beneficiaries with a primary diagnosis of acute ischemic stroke who were admitted to hospitals between January 1, 2009, and December 31, 2019, were included. The data analysis was performed between October 1, 2023, and September 9, 2024.

**Exposure:**

New certification of a stroke center within a 30-minute driving time of a community.

**Main Outcomes and Measures:**

The main outcomes were rates of admission to a certified stroke center, receipt of thrombolytics (delivered using drip-and-ship and drip-and-stay methods), mechanical thrombectomy, and 30-day and 1-year mortality estimated using a linear probability model with community fixed effects.

**Results:**

Among the 2 853 508 patients studied (mean [SD] age, 79.5 [8.5] years; 56% female), 66% lived in communities that had a stroke center nearby at baseline in 2009, and 34% lived in communities with no baseline access. For patients without baseline access, after stroke center expansion, the likelihood of admission to a stroke center increased by 38.98 percentage points (95% CI, 37.74-40.21 percentage points), and receipt of thrombolytics increased by 0.48 percentage points (95% CI, 0.24-0.73 percentage points). Thirty-day and 1-year mortality decreased by 0.28 percentage points (95% CI, −0.56 to −0.01) and 0.50 percentage points (95% CI, −0.84 to −0.15 percentage points), respectively, after expansion. For patients in communities with baseline stroke center access, expansion was associated with an increase of 9.37 percentage points (95% CI, 8.63-10.10 percentage points) in admission to a stroke center but no significant changes in other outcomes.

**Conclusions and Relevance:**

In this cohort study, patients living in communities without baseline stroke center access experienced significant increases in stroke center admission and thrombolysis and a significant decrease in mortality after a stroke center expansion. Improvements were smaller in communities with preexisting stroke center access. These findings suggest that newly certified stroke centers may provide greater benefits to underserved areas and are an important consideration when deciding when and where to expand health care services.

## Key Points

**Question:**

Do rates of admission to certified stroke centers, receipt of thrombolytic therapy or mechanical thrombectomy, and mortality change for patients with stroke when a hospital near their community becomes stroke certified?

**Findings:**

In this cohort study of more than 2.8 million patients, those living in communities without preexisting stroke center access experienced increases in stroke center admission and thrombolysis and a decrease in mortality after a stroke center expansion within a 30-minute drive. Improvements were smaller for communities with preexisting stroke center access at baseline.

**Meaning:**

These findings suggest that newly certified stroke centers may provide greater benefits to patients residing in areas previously without a nearby stroke center and that targeted efforts to support stroke center expansion in these areas may be necessary to improve patient outcomes.

## Teaser

This cohort study examines rates of admission, receipt of thrombolysis and mechanical thrombectomy, and mortality when a community gains a newly certified stroke center.

## Introduction

Stroke is a disease of aging, and risk doubles every decade for individuals older than 55 years.^1^ It is a leading cause of mortality among older adults and causes disability in 50% of survivors, leading to a loss of independence and, often, the need for long-term care.^2^ The timely administration of intravenous thrombolytic therapy^3^ has been recommended for eligible patients with stroke since 1995, and endovascular treatment with mechanical thrombectomy has been recommended since 2015.^4,5,6,7^ Both therapies have been associated with improvements in patient outcomes after stroke.^8,9^ The increased availability of acute stroke technology over the past decade offers a unique opportunity to examine how the rollout of stroke center certification has affected access to and delivery of these new treatments, as well as potential disparities in patient health outcomes.

Beginning in 2004, the American Heart Association, American Stroke Association, and The Joint Commission devised a process for certifying hospitals as primary stroke centers (PSCs),^10^ with the overall intention of improving medical care for patients with stroke. Since then, the number of certified stroke centers in the US (including more advanced tiers that specify additional capabilities^11^) has grown rapidly, from 964 hospitals (20.7% of all general acute hospitals) in 2009 to 2449 hospitals (54.2%) in 2022.^12^ It has been increasingly recognized that the infrastructure and institutional support a stroke center provides improves access to acute stroke treatments and subsequent patient care. Studies have shown that certified stroke centers are associated with timely treatment,^13^ decreased mortality risk,^14,15,16,17^ and improved functional outcomes^18^; however, the proliferation of these facilities has not occurred equally across the country. Hospitals serving low-income areas and those with lower profit margins,^19^ as well as segregated neighborhoods or those with a high share of Black residents,^20^ are much less likely to obtain stroke center certification than more affluent areas and predominantly White neighborhoods.

Despite rapid growth in the number of stroke centers over the past decade, there is little empirical evidence on the effects of this expansion at the population level. Geographic access is only one piece of the puzzle. Indeed, studies have shown that nearly 100% of the population has access to a hospital that can administer intravenous thrombolytics within 60 minutes,^21^ yet only 46% of patients with acute ischemic stroke who arrive at a hospital within 4.5 hours of presentation receive thrombolytics.^22^ Therefore, it is vital that we examine actual access, represented by receipt of care, rather than potential access, represented by geographic proximity. A nearby hospital could acquire stroke center certification, but patients might continue to receive care at alternate hospitals that are not stroke center certified because of nongeographic barriers (eg, patient preference, private transportation to hospital rather than emergency dispatch, etc). Stakeholders need empirical evidence to determine how the expansion of stroke centers affects stroke patients at the population level. To address this gap, we implemented a population-based approach to analyze changes in patients’ access to stroke centers, treatment (both thrombolysis and mechanical thrombectomy), and mortality when their community experienced an expansion in stroke center access, defined as a hospital within a 30-minute drive that acquired stroke center certification.

## Methods

### Data Sources

This cohort study was approved by the National Bureau of Economic Research. Informed consent was not required per the IRB Waiver of HIPAA Authorization, as approved by the National Bureau of Economic Research. We followed the Strengthening the Reporting of Observational Studies in Epidemiology (STROBE) reporting guideline.

We combined patient, hospital, and community-level data for this analysis. At the patient level, we used national 100% Medicare Provider and Analysis Review (MedPAR) files from 2009 through 2019 to identify the patient cohort and each patient’s community, as defined by their mailing zip code. At the hospital level, we collected stroke center certification data from national accrediting bodies and states (further details provided in the eMethods in Supplement 1).^23^ We supplemented stroke center certification data with data from the American Heart Association and the Healthcare Cost Report Information System to capture additional hospital characteristics, including geographic coordinates. At the community level, we used 2010 US census and 2011-2019 American Community Survey data to identify geographic coordinates and demographic information for each zip code community. Finally, we derived a driving-time database using web-based queries under normal traffic conditions from the geographic center of each patient’s zip code (where the geographic center was identified per US census) to stroke centers based on the geographic coordinates (longitude and latitude) of each location.^24^ We assumed normal traffic conditions for all queries because we did not have any information on when a patient was admitted to the hospital or their mode of transportation.

### Study Population

Our study population included all Medicare fee-for-service (FFS) beneficiaries with a primary diagnosis of acute ischemic stroke who were admitted to hospitals between January 1, 2009, and December 31, 2019. Based on previous literature, patients with stroke were identified by primary diagnosis using *International Classification of Diseases, Ninth Revision* (*ICD-9*) primary diagnosis codes 433.x1, 434.x1, or 436 or *International Statistical Classification of Diseases, Tenth Revision* (*ICD-10*) code I63.^25,26,27^ We omitted patients whose mailing zip code was more than 100 miles from their hospital admission as this likely indicated a discrepancy between their residential zip code and mailing zip code or that they received treatment while away from home.

### Identifying Hospital Stroke Center Status

Stroke centers were identified based on data from state and national certification programs. A hospital was identified as an acute stroke ready hospital (ASRH), a PSC, a thrombectomy-capable stroke center (TSC), or a comprehensive stroke center (CSC) on and after the year and quarter it achieved such status based on information from the national certification programs and state designation bodies. An ASRH represents the most basic level of stroke center certification and focuses on stabilizing the patient and initiating emergency stroke treatment. In most cases, patients treated at an ASRH are transferred to a more advanced facility for further care. A PSC delivers organized inpatient stroke care, and TSCs and CSCs are specifically recognized for having the additional capacity to perform mechanical thrombectomy. In our main analysis, we defined a hospital as a stroke center if it achieved any level of stroke center certification. In our sensitivity analysis, we stratified certification levels into 3 tiers: ASRH, PSC, and TSC or CSC.

### Identifying Baseline and Changes to Stroke Center Access at the Community Level

In our statistical models, we identified each community’s baseline stroke center access to investigate differential outcomes associated with baseline differences in access. A community was classified as having no baseline stroke center access if there was no certified stroke center (at any level) within a 30-minute drive (alternately referred to as nearby) by the first quarter of 2009. A community was classified as having baseline stroke center access if there was at least 1 nearby stroke center by the first quarter of 2009.

We then identified changes in stroke center access as follows: For a given community, the change indicator became 1 on and after the year-quarter that a nearby hospital acquired stroke center certification. These measures were constructed using the following steps. First, using our driving-time database, we identified all hospitals within a 30-minute drive of the geographic center of each zip code community for each year-quarter, regardless of the hospital’s stroke center status. Then, for each set, we evaluated quarter-to-quarter changes in hospital stroke certification status and classified communities according to whether a nearby hospital acquired stroke center certification. We chose a threshold travel time of 30 minutes given the time-sensitive nature of efficacy and risks associated with acute stroke interventions, as well as previous studies of travel times for medical treatments, including emergency cesarean delivery,^28^ cardiac care,^29,30^ and primary care.^31^

### Outcomes

We evaluated 3 types of outcomes: (1) access, defined as whether a patient with stroke was admitted to a certified stroke center regardless of certification level; (2) receipt of treatment, defined as thrombolysis and mechanical thrombectomy based on *ICD-9* and *ICD-10* procedure codes^32,33,34^; and (3) mortality, defined as short-term (30-day) or long-term (1-year) mortality. In our sensitivity analysis, we further separated patients who received thrombolytic therapy into 2 categories: (1) drip and ship, when intravenous thrombolysis was initiated in the emergency department at 1 facility and the patient was emergently transferred to another facility for further care, and (2) drip and stay, when the patient received intravenous thrombolysis at the same facility for inpatient care.

### Statistical Analysis

The data analysis was performed between October 1, 2023, and September 9, 2024. Our goal was to compare changes in outcomes when a community experienced an expansion in stroke center access (treatment communities) with communities that did not experience a change in stroke center access (reference communities). Our empirical model followed a difference-in-differences framework. Because our outcomes were binary, we implemented a linear probability model with community fixed effects. Although a probit or logit model is a natural choice for estimating a dichotomous variable in cross-sectional data, these models result in inconsistent estimators in panel data with a large number of community fixed effects.^35^ The linear probability model can consistently estimate the association of stroke access change with dichotomous outcomes.^36^ In our main model, the key variables to capture stroke expansion assumed the value 1 on and after the year-quarter that a community experienced a stroke center expansion within a 30-minute drive. For all analyses, we estimated this model separately for patients in communities with and without existing stroke center access at baseline. The coefficients of the key variables represent changes in the outcome’s probability after a hospital acquires stroke center certification within 30 minutes of a treatment community compared with the same probability in the control community that has no change in stroke center access. These community fixed effects are crucial because they control for any unobserved time-invariant heterogeneity across communities, including any baseline differences in underlying patient health and socioeconomic conditions. We included time dummies to control for secular trends as well as patient demographic covariates (race and ethnicity, sex, 5-year age groups) and disease-related risk adjustments, following prior work.^37,38^ Self-reported race and ethnicity (Black, Hispanic, White, other [including Asian, Native American, other not mentioned before, unknown^39^]) were collected from the MedPAR files and included to elucidate patient demographic information for each community type.

Our main analysis focused on overall associations by grouping all certified stroke centers together. In our sensitivity analysis, we analyzed potential differences in treatment and health outcomes based on whether the highest level of certified stroke center nearby was ASRH, PSC, or TSC or CSC. Data were analyzed using Stata, version 18 (StataCorp LLC). A 2-sided *P* < .05 by *t* test was considered significant.

## Results

**Figure 1.**
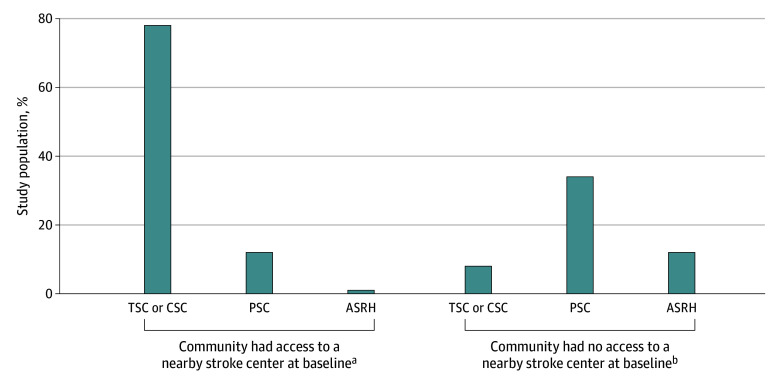
Percentage of Patients Exposed to Newly Certified Stroke Centers by Highest Certification Level, 2009-2019 Based on the equality of proportions test, the pairwise comparison between the share of patients who were exposed to a thrombectomy-capable stroke center (TSC) or comprehensive stroke center (CSC) was statistically significantly different from the share of patients who were exposed to the other 2 levels of stroke certifications (acute stroke ready hospital [ASRH] and primary stroke center [PSC]) for both types of communities (stroke center nearby at baseline, 78% vs 13% [*P* < .001]; stroke center not nearby at baseline, 8% vs 46% [*P* < .001]). ^a^n = 1 896 110. ^b^n = 957 176.

Our final analytic cohort consisted of 2 853 508 patients (mean [SD] age, 79.5 [8.5] years; 56% female and 44% male; 13% Black, 2% Hispanic, 81% White, and 2% other race or ethnicity [including Asian, American Indian or Alaska Native, other, or unknown]) living in 36 209 zip code communities over the 11-year study period. Figure 1 shows that among the 957 176 patients (34% of the study population) living in communities with no nearby stroke center at baseline, 54% were exposed to a stroke center expansion nearby (12% to a newly certified ASRH, 34% to a PSC, and 8% to a TSC or CSC). In contrast, among the 1 896 110 patients (66% of the study population) who lived in communities that already had stroke center access at baseline, 91% were exposed to a newly certified stroke center nearby (1% to an ASRH, 12% to a PSC, and 78% to a TSC or CSC). By the end of the study period in 2019, 17% of patients in our study population lived in communities that still did not have a nearby stroke center, and in communities that experienced an expansion in available stroke centers, 8% of patients continued to be admitted to nonstroke centers in 2019. The Table provides patient characteristics from 2009 to 2019 stratified by baseline geographic stroke center access in 2009. eTable 1 in Supplement 1 reports mean patient access, treatment, and outcomes at baseline in 2009. Patients in communities with no baseline access were more likely to be White (87% vs 78%) and were predominantly located in rural areas (55% vs 3%).

**Table.** Descriptive Statistics of Patient Characteristics Between 2009 and 2019, Stratified by Baseline Stroke Center Availability in 2009

Characteristic	No. of patients (%)			*P* value of mean differences
	Total sample	No stroke center nearby at baseline in 2009^a^	Preexisting stroke center nearby at baseline in 2009^a^	
No. of patients	2 853 508	957 176	1 896 110	
Race and ethnicity				
Black	371 054 (13)	89 623 (9)	281 411 (15)	<.001
Hispanic	56 019 (2)	11 205 (1)	44 812 (2)	<.001
White	2 313 913 (81)	834 504 (87)	1 479 215 (78)	<.001
Other^b^	56 202 (2)	10 864 (1)	45 333 (2)	<.001
Sex				
Female	1 598 826 (56)	525 258 (55)	1 073 437 (57)	<.001
Male	1 254 682 (44)	431 918 (45)	822673 (43)	<.001
Age distribution at time of admission, y				
65-69	451 664 (16)	159 951 (17)	291 699 (15)	<.001
70-74	481 049 (17)	172 844 (18)	308 190 (16)	<.001
75-79	511 009 (18)	180 788 (19)	330 206 (17)	<.001
80-84	535 264 (19)	179 859 (19)	355 358 (19)	.32
≥85	874 522 (31)	263 734 (28)	610 657 (32)	<.001
Living in rural area	592 770 (21)	527 396 (55)	65 374 (3)	<.001
Patient conditions				
Recurring stroke	244 045 (9)	76 921 (8)	167 098 (9)	<.001
Transferred to another hospital	215 073 (8)	128 843 (13)	86 216 (5)	<.001
Peripheral vascular disease	282 771 (10)	90 599 (9)	192 158 (10)	<.001
Pulmonary circulation disorders	98 283 (3)	31 063 (3)	67 210 (4)	<.001
Diabetes	935 697 (33)	317 620 (33)	618 029 (33)	<.001
Kidney failure	531 686 (19)	170 682 (18)	360 961 (19)	<.001
Liver	28 181 (1)	8643 (1)	19 537 (1)	<.001
Cancer	113 770 (4)	36 054 (4)	77 704 (4)	<.001
Dementia	301 969 (11)	93 656 (10)	208 277 (11)	<.001
Valvular disease	273 525 (10)	85 526 (9)	187 970 (10)	<.001
Hypertension	2 422 194 (85)	806 858 (84)	1 615 154 (85)	<.001
Chronic pulmonary disease	456 130 (16)	163 845 (17)	292 246 (15)	<.001
Rheumatoid arthritis or collagen vascular disease	78 346 (3)	26 244 (3)	52 096 (3)	.78
Coagulation deficiency	106 548 (4)	33 070 (3)	73 472 (4)	<.001
Obesity	232 771 (8)	80 405 (8)	152 357 (8)	<.001
Substance use	62 031 (2)	20 416 (2)	41 613 (2)	.001
Depression	274 208 (10)	94 026 (10)	180 161 (10)	<.001
Psychosis	191 641 (7)	62 754 (7)	128 877 (7)	<.001
Hypothyroidism	493 026 (17)	168 199 (18)	324 785 (17)	<.001
Paralysis and other neurologic disorder	1 640 978 (58)	552 109 (58)	1 088 738 (57)	<.001
Ulcer	9748 (0)	3194 (0)	6554 (0)	.10
Weight loss	126 442 (4)	41 154 (4)	85 267 (4)	<.001
Fluid and electrolyte disorders	652 229 (23)	215 877 (23)	436 309 (23)	<.001
Anemia (blood loss and deficiency)	357 508 (13)	112 244 (12)	245 230 (13)	<.001
Patient access, treatment, and health outcomes				
Admitted to stroke center	2 181 584 (76)	550 958 (58)	1 630 462 (86)	<.001
Received thrombolytic therapy during hospitalization	285 514 (10)	90 337 (9)	195 153 (10)	<.001
Received mechanical thrombectomy during hospitalization	75 875 (3)	22 170 (2)	53 702 (3)	<.001
30-d Mortality	377 206 (13)	132 026 (14)	245 137 (13)	<.001
1-y Mortality	806 641 (28)	274 559 (29)	531 993 (28)	<.001

^a^ Access at baseline is defined as having a certified stroke center within 30 minutes before 2009.

^b^ Other race and ethnicity included Asian, American Indian or Alaska Native, other, or unknown.

**Figure 2.**
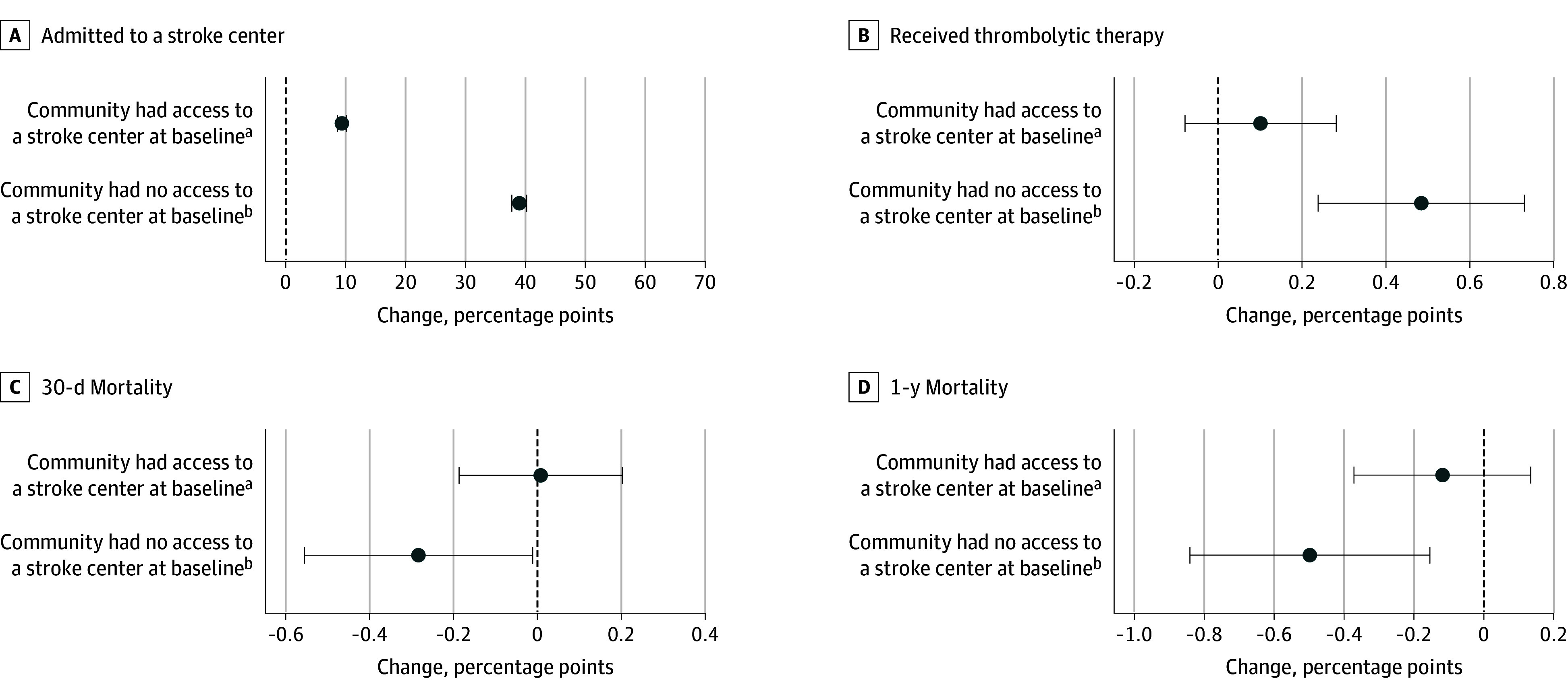
Changes in Patient Access, Treatment, and Mortality Rates When a Hospital Within a 30-Minute Drive Gained Stroke Certification Access at baseline is defined as having a certified stroke center within 30 minutes before 2009. Error bars indicate the 95% CIs. ^a^n = 1 896 110. ^b^n = 957 176.

Figure 2 (complete results provided in eTable 2A and B in Supplement 1) shows risk-adjusted changes in patient outcomes when a community gained a certified stroke center nearby vs a community with similar baseline geographic access that did not experience a change in stroke center access during the study period (reference community). The probability of being admitted to a stroke center increased by 38.98 percentage points (95% CI, 37.74-40.21 percentage points) from a base rate of 23.94% (eTable 1 in Supplement 1) when a hospital in a community with no baseline stroke center access acquired stroke certification within a 30-minute drive compared with the reference community (Figure 2A). The improvement in actual access was smaller (9.37 percentage points; 95% CI, 8.63-10.10 percentage points) if the community had baseline stroke center access.

Figure 2B shows that if a newly certified stroke center was introduced near a community with no baseline stroke center access, the probability of receiving thrombolytic therapy increased by 0.48 percentage points (95% CI, 0.24-0.73 percentage points) from a base rate of 3.83% (eTable 1 in Supplement 1). In contrast, if the newly certified hospital was near a community with preexisting baseline stroke center access, there was no significant change in the probability of receiving thrombolytic therapy.

The results presented in eTable 2A and B in Supplement 1 further differentiate between 2 different models of delivering thrombolytic therapy (ie, drip and ship, drip and stay). For communities with no baseline stroke center access, the proportion of patients receiving drip-and-stay therapy increased by 0.34 percentage points (95% CI, 0.13-0.54 percentage points), while there were no significant changes in those treated with the drip-and-ship model. In communities that already had stroke center access at baseline, no significant changes in either mode of delivering thrombolytic therapy were observed. Furthermore, the introduction of a newly certified stroke center did not significantly change the probability of receiving mechanical thrombectomy in either community type.

Finally, Figure 2C and D show that in communities with no baseline stroke center access, 30-day and 1-year mortality decreased by 0.28 percentage points (95% CI, −0.56 to −0.01 percentage points) and 0.50 percentage points (95% CI, −0.84 to −0.15 percentage points), respectively, when a newly certified stroke center was introduced nearby. In communities with stroke center access at baseline, no significant changes in either mortality outcome were observed when an additional stroke center was certified nearby.

**Figure 3.**
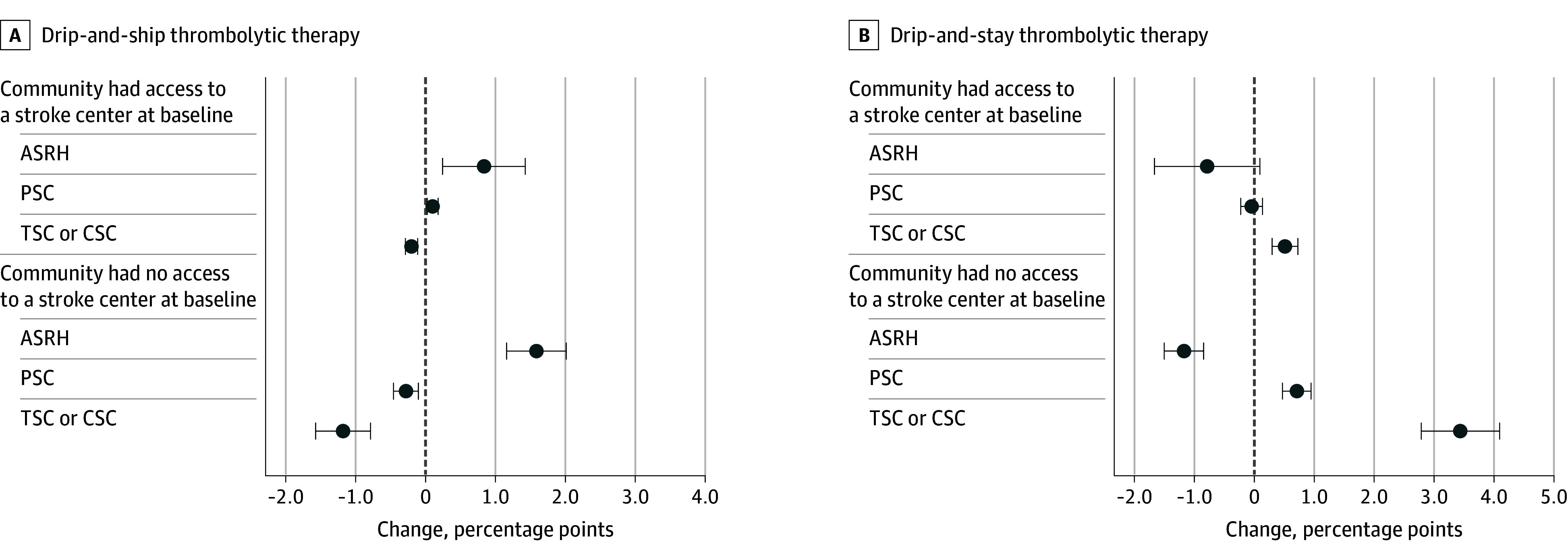
Changes in Drip-and-Ship and Drip-and-Stay Thrombolytic Therapy Rates When a Hospital Within a 30-Minute Drive Gained Stroke Certification, by Highest Certification Level Acquired Access at baseline is defined as having a certified stroke center within 30 minutes before 2009. Error bars indicate 95% CIs. ASRH indicates acute stroke ready hospital; CSC, comprehensive stroke center; PSC, primary stroke center; TSC, thrombectomy-capable stroke center.

In our sensitivity analysis, we further investigated whether changes in treatment patterns were driven by the level of stroke center certification. Specifically, we enhanced our main model to allow the coefficients to differ between communities where the highest new certification level of a stroke center was ASRH, PSC, or TSC or CSC (Figure 3; eTable 3 in Supplement 1). For communities with baseline stroke center access, a newly certified ASRH or PSC was associated with an increase of 0.83 percentage points (95% CI, 0.24-1.43 percentage points) and 0.10 percentage points (95% CI, 0.02-0.18 percentage points), respectively, in patients receiving drip-and-ship therapy (Figure 3A), while there were no significant changes in those receiving drip-and-stay therapy (Figure 3B). However, a newly certified TSC or CSC was associated with a decrease of 0.20 percentage points (95% CI, −0.29 to −0.11 percentage points) in patients receiving drip-and-ship therapy and an increase of 0.47 percentage points (95% CI, 0.26-0.69 percentage points) in those receiving drip-and-stay therapy. For communities without a nearby stroke center at baseline, a newly certified ASRH was associated with a significant increase in patients receiving drip-and-ship therapy (1.58 percentage points; 95% CI, 1.16-2.01 percentage points) (Figure 3A) and a decrease in those receiving drip-and-stay therapy (−1.17 percentage points; 95% CI, −1.50 to −0.84 percentage points) (Figure 3B). A new PSC was associated with a decrease in patients receiving drip-and-ship therapy (0.28 percentage points; 95% CI, −0.46 to −0.10 percentage points) and an increase in those receiving drip-and-stay therapy (0.71 percentage points; 95% CI, 0.47-0.95 percentage points). A newly certified TSC or CSC was also associated with a decrease in patients receiving drip-and-ship therapy (−1.18 percentage points; 95% CI, −1.57 to −0.79 percentage points) and an increase in those receiving drip-and-stay therapy (3.44 percentage points; 95% CI, 2.79-4.09 percentage points).

**Figure 4.**
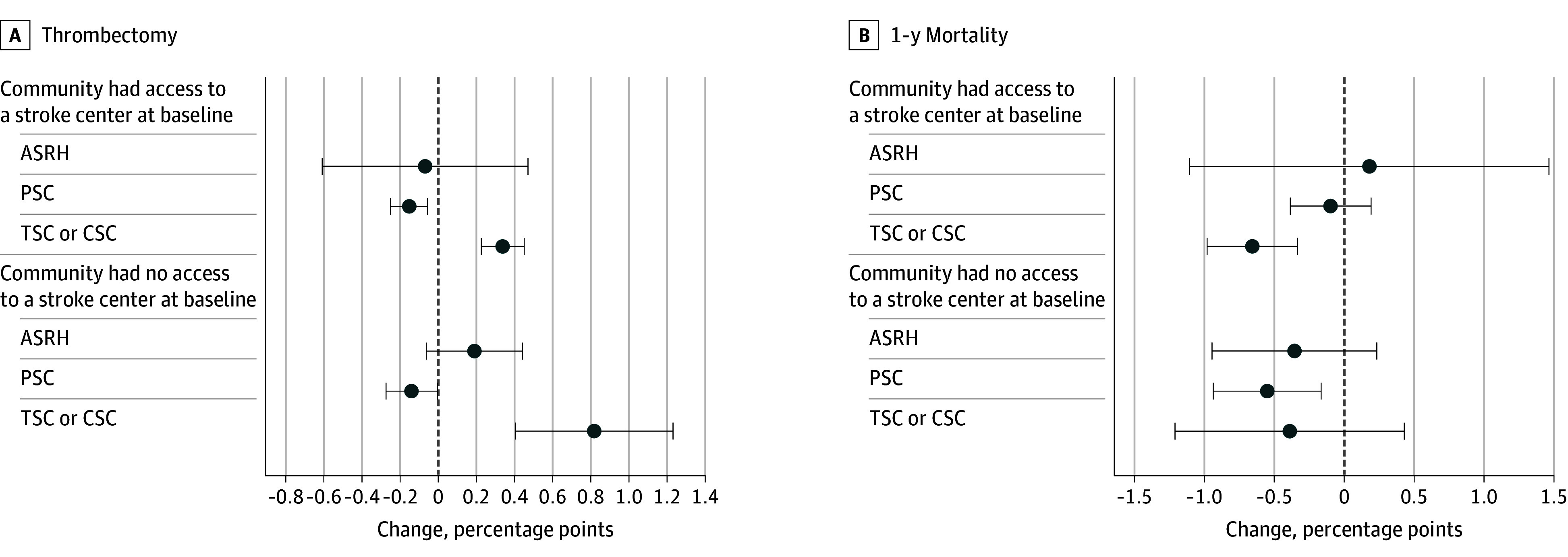
Changes in Thrombectomy and 1-Year Mortality Rates When a Hospital Within a 30-Minute Drive Gained Stroke Certification, by Highest Certification Level Acquired Access at baseline is defined as having a certified stroke center within 30 minutes before 2009. Error bars indicate 95% CIs. ASRH indicates acute stroke ready hospital; CSC, comprehensive stroke center; PSC, primary stroke center; TSC, thrombectomy-capable stroke center.

Figure 4 shows that the introduction of newly certified stroke centers specifically recognized for mechanical thrombectomy capabilities (ie, TSCs, CSCs) increased the likelihood that patients in those communities received that treatment. For communities with baseline stroke center access, an increase of 0.34 percentage points (95% CI, 0.23-0.45 percentage points) in mechanical thrombectomy was observed, whereas an increase of 0.82 percentage points (95% CI, 0.41-1.23 percentage points) was seen for communities with no baseline access (Figure 4A). Expansion of other levels of stroke center certification (ie, PSCs, ASRHs) had a negative or null association with the likelihood of receiving mechanical thrombectomy. Finally, the reduction in mortality from our main model appears to be driven by the certification of new TSCs and CSCs in communities with preexisting stroke capacity, and by PSCs in communities with no baseline access (Figure 4B). Recall that TSCs and CSCs are the most common type of stroke center introduced in communities with preexisting stroke center capacity, whereas PSCs are the most commonly introduced in communities with no baseline capacity (Figure 1).

## Discussion

In our 11-year cohort study of more than 2.8 million patients with stroke, we found that among the 34% of patients in communities without preexisting geographic access to stroke centers at baseline in 2009, 54% were exposed to a newly certified stroke center nearby during the study period; among the 66% who lived in communities with a nearby stroke center prior to 2009, 91% were exposed to additional certified stroke centers. In communities without baseline access, stroke center expansion increased the probability of admission to a certified stroke center by 38.98 percentage points, a 160% relative increase, given the base rate of 23.94% in this community type. Communities with no baseline access also experienced a 0.48-percentage point increase, or 13% relative increase, in receipt of thrombolytic therapy (base rate of 3.83%), and improvements varied by the highest level of stroke certification obtained. Communities with newly certified ASRHs were more likely to experience an increase in the delivery of drip-and-ship therapy, while those with new PSCs, TSCs, or CSCs experienced an increase in delivery of drip-and-stay therapy. While we did not observe an overall change in the receipt of mechanical thrombectomy, there was an increase of 0.82 percentage points (equivalent to a 160% relative increase) in thrombectomy if the exposure was to a newly certified TSC or CSC. Finally, when communities without baseline stroke access were exposed to a newly certified stroke center, we found a small but significant improvement in 30-day and 1-year mortality (0.28 and 0.50 percentage points, respectively, or a 2% relative decrease). While exposure to newly certified stroke hospitals increased the probability of being admitted to a stroke center among patients in communities with baseline stroke center access by 13%, such exposure did not change their treatment and health outcomes.

Our main finding, that new stroke centers are associated with greater benefits when introduced near communities without baseline geographic access, echoes findings from prior literature on the distribution of health care services for other time-sensitive conditions. For instance, patients with acute myocardial infarction residing in average-capacity markets experience significantly greater benefits after the introduction of new percutaneous coronary intervention services near their community than those in high-capacity markets.^40^ Our study suggests that the same may be true for specialized stroke services. Certified stroke centers, particularly those with advanced certifications (PSC, TSC, and CSC), are more likely to cluster in geographic areas with high population density and proximity to academic institutions.^19,41^ If sufficient services are already available nearby, the introduction of a new stroke center may not yield additional benefits.

Furthermore, the mechanism through which stroke center expansion and corresponding changes in outcomes occur differs based on the level of stroke center introduced. In communities with no baseline access (mostly in rural regions), 12% of patients gained a newly certified ASRH nearby while just 8% gained a TSC or CSC. In contrast, 78% of patients in communities with baseline stroke center access gained a newly certified TSC or CSC during the study period. Moreover, newly certified ASRHs were associated with increased proportions of patients receiving drip-and-ship therapy and decreased proportions receiving drip-and-stay therapy, while new PSCs, TSCs, and CSCs largely showed the opposite. Given the intended role of ASRHs to serve predominantly rural or remote communities and deliver initial stroke treatment and the role of PSCs, TSCs, and CSCs to deliver more advanced care, it is reassuring to see that stroke center expansion over the past decade has produced many of the desired benefits. For ASRHs in particular, stroke center certification may be indicative of benefits beyond certification of the individual hospital. The significant decrease in the proportion of patients receiving drip-and-stay therapy after the introduction of a new ASRH in communities with no existing stroke center access may reflect improved integration of that ASRH into a regional stroke network or advancements in local, regional, or state stroke systems of care. On the other hand, our results also illustrate that although newly certified TSCs and CSCs increased receipt of thrombolytics and mechanical thrombectomy, in accordance with prior literature,^42^ very little TSC and CSC expansion occurred near communities without baseline access. This lack of expansion may lead to growing disparities in receipt of these critical treatments, particularly for mechanical thrombectomy, which TSCs and CSCs are specifically recognized for having the capability to perform.

Our findings have several implications. First, stroke center expansion over the past decade has produced many of the desired benefits. The expansion of newly certified stroke centers was associated with increased admissions to stroke centers. At the population level, meaningful changes in thrombolysis (and mechanical thrombectomy when hospitals with those capabilities were nearby) were also observed, and patterns were consistent with the intent of the certification program (eg, increased delivery of drip-and-ship therapy in remote communities and drip-and-stay therapy in urban communities). These findings suggest that certification efforts are largely working as intended, with patients with stroke being preferentially routed to stroke centers and possibly receiving more timely and improved care, conditional on being admitted to a stroke center.^43^ In light of these findings, it is important to remember that stroke certification is granted after capacity has been in place for some time, meaning that the hospitals have already shown the capability to care for patients with stroke (especially for TSCs and CSCs where minimum volumes are required for certification) before the certification is granted. Because our study uses the year of actual certification to examine the association of certification with patient outcomes, our estimates are therefore conservative. In other words, the actual advantages of certification may surpass what was observed in this study.

It is also important to note that stroke center certification is not an arbitrary decision or process. While stroke centers have continued to proliferate in recent years (from 2223 stroke centers in 2019 to 2449 in 2022^12^), coverage remains inequitable. Expansion has disproportionately occurred in socioeconomically advantaged communities, and hospitals in disadvantaged and rural communities are much less likely to be certified.^12^ While it is reassuring to see that the introduction of a newly certified stroke center near a community improves stroke care for that population, 17% of patients in our study population lived in communities that still did not have a stroke center within a 30-minute drive by the end of the study period in 2019. Furthermore, even in communities that experienced an expansion in available stroke centers, 8% of patients continued to be admitted to nonstroke centers in 2019.

While certification does not always require a major infrastructure investment (eg, a cardiac catheterization laboratory as a hub for receiving patients with ST-elevation myocardial infarction, trauma surgeons for a level I trauma center), depending on the certification level, stroke center certification requires a commitment to reporting and process improvement based on standardized metrics and written protocols, and centers must meet education and staffing standards, all of which require resources. These resource requirements,^11^ taken together with our findings, suggest that implementing targeted incentives and support for hospitals in communities without existing stroke center access may be a critical factor for expanding certification and, thus, improving outcomes for patients with stroke. There may also be some cause for concern regarding service expansion in more affluent communities with preexisting access to these services. Prior literature has shown that service expansion for other time-sensitive conditions (eg, acute myocardial infarction) may not produce the desired benefits and may even be associated with reduced volumes and adverse patient outcomes.^44,45,46,47^

### Limitations

This study has several limitations. First, our patient population did not include patients with stroke outside the Medicare FFS program. However, our working assumption is that the paths through which stroke care expansion are correlated with patient outcomes are similar between Medicare FFS and non–Medicare FFS beneficiaries.^48^ As such, our findings may still inform overall policy design and certification guidelines. Second, because MedPAR is an administrative dataset, it provides limited clinical information, which precluded us from identifying the severity of stroke in each patient. However, MedPAR is the only longitudinal national dataset that supplies community information for individual patients, so avoiding this limitation was not possible. Third, driving time measures were imprecise, as we used the same geographic coordinates for all patients from a given community and assumed normal traffic conditions for all driving time queries. This imprecision would bias our results toward zero, rendering our findings a conservative estimate. Finally, we were limited to mortality outcomes since we did not have access to more granular clinical data to analyze additional functional outcomes (eg, ability to perform independent activities of daily living). Because such outcomes are known to be more sensitive to changes in receipt of quality stroke care, our findings are therefore conservative.

## Conclusions

In this cohort study, we found that patients in communities without geographic access to a stroke center at baseline in 2009 experienced a 160% increase in stroke center admission, a 13% increase in thrombolysis, and a 2% reduction in mortality, while improvements were smaller or not significant in communities with preexisting stroke center access at baseline. These findings suggest that the benefits of a newly certified stroke center may be much greater in underserved areas and are an important consideration when deciding when and where to expand health care services.

## Notes

Supplement 1. **eMethods.** Stroke Center Data Collection Process **eTable 1.** Mean Patient Access, Treatment, and Health Outcomes at Baseline in 2009, Stratified by Baseline Stroke Center Availability **eTable 2A.** Full Regression Results of the Models Presented in Figure 2 for Communities With Baseline Access to a Certified Stroke Center **eTable 2B.** Full Regression Results of the Models Presented in Figure 2 for Communities With No Baseline Access to a Certified Stroke Center **eTable 3.** Risk-Adjusted Percentage Point Changes in Treatment and Mortality Outcomes When a Newly Certified Stroke Center Becomes Available Within a 30-Minute Drive, Stratified by Baseline Stroke Center Access and Highest Certification Level

Supplement 2.
Data Sharing Statement
