# Supplementary material for: Treatments and Patient Outcomes Following Stroke Center Expansion
Source: JAMA Netw Open. 2024 Nov 13;7(11):e2444683. doi: 10.1001/jamanetworkopen.2024.44683 (PMC11561690; doi:10.1001/jamanetworkopen.2024.44683)
Supplement: Supplement 1. — eMethods. Stroke Center Data Collection Process eTable 1. Mean Patient Access, Treatment, and Health Outcomes at Baseline in 2009, Stratified by Baseline Stroke Center Availability eTable 2A. Full Regression Results of the Models Presented in Figure 2 for Communities With Baseline Access to a Certified Stroke Center eTable 2B. Full Regression Results of the Models Presented in Figure 2 for Communities With No Baseline Access to a Certified Stroke Center eTable 3. Risk-Adjusted Percentage Point Changes in Treatment and Mortality Outcomes When a Newly Certified Stroke Center Becomes Available Within a 30-Minute Drive, Stratified by Baseline Stroke Center Access and Highest Certification Level [file jamanetwopen-e2444683-s001.pdf]

Supplemental Online Content

Shen Y-C, Kim AS, Hsia RY. Treatments and patient outcomes following stroke center expansion. *JAMA Netw Open*. 2024;7(11):e2444238. doi:10.1001/jamanetworkopen.2024.44238

eMethods. Stroke Center Data Collection Process

eTable 1. Mean Patient Access, Treatment, and Health Outcomes at Baseline in 2009, Stratified by Baseline Stroke Center Availability

eTable 2A. Full Regression Results of the Models Presented in Figure 2 for Communities With Baseline Access to a Certified Stroke Center

eTable 2B. Full Regression Results of the Models Presented in Figure 2 for Communities With No Baseline Access to a Certified Stroke Center

eTable 3. Risk-Adjusted Percentage Point Changes in Treatment and Mortality Outcomes When a Newly Certified Stroke Center Becomes Available Within a 30-Minute Drive, Stratified by Baseline Stroke Center Access and Highest Certification Level

This supplemental material has been provided by the authors to give readers additional information about their work.

© 2024 Shen Y-C et al. *JAMA Network Open.*

eMethods. Stroke Center Data Collection Process

We undertook a multi-step process to ensure we accurately captured the U.S. stroke center landscape. To identify stroke centers certified by one of the four Centers for Medicare and Medicaid Services-approved certifying organizations, we either a) identified a publicly available list of certified stroke centers on the organization's webpage (i.e., The Joint Commission), or b) contacted a representative at the organization to acquire a list of current and historical stroke centers (i.e., Det Norske Veritas, Accreditation Commission for Health Care, and Center for Improvement in Healthcare Quality). We also identified 14 states that offered an independent state process for stroke center certification (i.e., stroke centers could be self-certified/independently designated by the state without a national certification). To identify these self-certified/independently designated stroke centers, we took the following steps:

1. From September to October 2022, we conducted internet searches of public-facing webpages and administered a survey instrument to PIs at the 25 National Institutes of Health StrokeNet Regional Coordinating Centers around the country to obtain preliminary information on state certification or designation offerings.
2. We proceeded to categorize states into two groups: states with a self-certification or independent designation process and those without.
3. From October 2022 through April 2023 we verified the preliminary categorizations of state processes from Step 3 using peer-reviewed literature, primary source documents from states (e.g., state policy documents or legislation), and/or direct contact with state officials.
4. For states preliminarily determined to have a self-certification or independent designation process, we confirmed with a state official whether there was an independent state

© 2024 Shen Y-C et al. *JAMA Network Open.*

process and requested a current and historical list of stroke centers that had been self-certified/independently designated by the state.

5. For states preliminarily determined not to have a self-certification or independent designation process, we confirmed that there was, in fact, no self-certification or independent designation process. If our preliminary categorization was incorrect and an independent state designation option was available, we followed the process outlined in Step 4.

© 2024 Shen Y-C et al. *JAMA Network Open.*

eTable 1. Mean Patient Access, Treatment, and Health Outcomes at Baseline in 2009, Stratified by Baseline Stroke Center Availability

	Whole Sample		No stroke center nearby at baseline in 2009		Pre-existing stroke center nearby at baseline in 2009		p-value of mean differences
	N	%	N	%	N	%	
**Patients**	225,125		74,284		150,806		
Admitted to stroke center	125,980	56.0%	17,784	23.9%	108,173	71.7%	<0.001
Received thrombolytic therapy during hospitalization	11,211	5.0%	2,845	3.8%	8,355	5.5%	<0.001
Received mechanical thrombectomy during hospitalization	1,643	0.7%	394	0.5%	1,252	0.8%	<0.001
30-day mortality	32,238	14.3%	11,209	15.1%	21,022	13.9%	<0.001
1-year mortality	66,930	29.7%	22,493	30.3%	44,412	29.5%	<0.001

© 2024 Shen Y-C et al. *JAMA Network Open.*

eTable 2A. Full Regression Results of the Models Presented in Figure 2 for Communities With Baseline Access to Certified Stroke Centers

	Admitted to any stroke center	Thrombolytics (overall)	Drip-and-ship	Drip-and-stay	Mechanical thrombectomy	30-day mortality	1-year mortality
on and after year-quarter gaining stroke center within 30-min	9.37**	0.10	0.03	0.07	-0.01	0.01	-0.12
	[8.63,10.10]	[-0.08,0.28]	[-0.04,0.11]	[-0.10,0.24]	[-0.10,0.09]	[-0.19,0.20]	[-0.37,0.13]
Black	-0.46**	-2.07**	-0.36**	-1.71**	-0.68**	-3.38**	-2.10**
	[-0.68,-0.23]	[-2.23,-1.92]	[-0.43,-0.30]	[-1.85,-1.56]	[-0.76,-0.59]	[-3.53,-3.22]	[-2.33,-1.87]
Hispanic	-1.19**	-0.37*	-0.08	-0.29*	0.04	-1.02**	-1.92**
	[-1.57,-0.81]	[-0.68,-0.07]	[-0.20,0.03]	[-0.57,-0.01]	[-0.14,0.22]	[-1.34,-0.70]	[-2.34,-1.49]
Other Race	-0.69**	-0.76**	0.03	-0.79**	0.17	-1.61**	-2.76**
	[-1.01,-0.37]	[-1.06,-0.46]	[-0.10,0.16]	[-1.07,-0.51]	[-0.02,0.35]	[-1.87,-1.34]	[-3.12,-2.40]
Female	-0.31**	-0.37**	-0.00	-0.37**	0.17**	1.17**	0.43**
	[-0.40,-0.21]	[-0.46,-0.28]	[-0.04,0.04]	[-0.45,-0.28]	[0.12,0.23]	[1.08,1.27]	[0.30,0.56]
Age 70-74	0.01	-0.02	0.01	-0.03	0.08	0.36**	0.30**
	[-0.15,0.17]	[-0.18,0.13]	[-0.06,0.07]	[-0.17,0.11]	[-0.01,0.17]	[0.24,0.48]	[0.12,0.47]
Age 75-79	-0.07	-0.07	-0.01	-0.07	0.06	1.79**	3.93**
	[-0.23,0.09]	[-0.23,0.09]	[-0.07,0.06]	[-0.21,0.08]	[-0.03,0.15]	[1.66,1.92]	[3.74,4.11]
Age 80-84	-0.16	-0.94**	-0.22**	-0.72**	-0.27**	4.52**	9.43**
	[-0.32,0.01]	[-1.09,-0.78]	[-0.28,-0.15]	[-0.86,-0.57]	[-0.36,-0.19]	[4.37,4.66]	[9.23,9.63]
Age 85+	-0.52**	-1.97**	-0.49**	-1.47**	-1.03**	13.48**	24.36**
	[-0.68,-0.35]	[-2.11,-1.82]	[-0.55,-0.43]	[-1.61,-1.34]	[-1.12,-0.95]	[13.34,13.63]	[24.16,24.56]
Recurrent stroke	0.16*	-2.25**	-0.57**	-1.68**	-0.80**	1.77**	5.10**
	[0.01,0.31]	[-2.40,-2.10]	[-0.63,-0.51]	[-1.81,-1.54]	[-0.88,-0.72]	[1.60,1.94]	[4.87,5.33]
Peripheral vascular disease	1.01**	-0.38**	-0.10**	-0.28**	0.42**	0.32**	1.71**
	[0.82,1.21]	[-0.52,-0.24]	[-0.16,-0.04]	[-0.42,-0.15]	[0.33,0.50]	[0.16,0.48]	[1.50,1.92]

© 2024 Shen Y-C et al. *JAMA Network Open.*

Pulmonary circulation disorders	0.99**	1.34**	0.12*	1.22**	1.13**	4.35**	8.30**
	[0.73,1.25]	[1.10,1.59]	[0.02,0.22]	[0.99,1.45]	[0.99,1.28]	[4.04,4.65]	[7.93,8.67]
Diabetes	-0.26**	-1.72**	-0.33**	-1.39**	-0.67**	-0.07	1.52**
	[-0.37,-0.16]	[-1.82,-1.63]	[-0.37,-0.29]	[-1.48,-1.30]	[-0.72,-0.62]	[-0.17,0.03]	[1.39,1.65]
Kidney failure	0.01	-1.39**	-0.45**	-0.94**	-0.87**	2.52**	8.01**
	[-0.12,0.14]	[-1.51,-1.28]	[-0.49,-0.40]	[-1.05,-0.84]	[-0.93,-0.81]	[2.39,2.65]	[7.84,8.18]
Liver disease	0.32	-2.37**	-0.64**	-1.72**	-0.95**	2.05**	5.96**
	[-0.12,0.75]	[-2.76,-1.97]	[-0.80,-0.49]	[-2.10,-1.35]	[-1.19,-0.70]	[1.57,2.52]	[5.33,6.59]
Cancer	0.34**	-3.23**	-0.62**	-2.61**	-0.37**	13.82**	30.40**
	[0.11,0.56]	[-3.42,-3.03]	[-0.70,-0.54]	[-2.79,-2.43]	[-0.49,-0.24]	[13.51,14.14]	[30.04,30.75]
Dementia	-1.41**	-3.73**	-0.83**	-2.90**	-2.08**	3.56**	11.63**
	[-1.57,-1.26]	[-3.86,-3.59]	[-0.88,-0.77]	[-3.03,-2.77]	[-2.15,-2.00]	[3.38,3.74]	[11.39,11.87]
Valvular disease	0.28**	0.03	-0.15**	0.18**	0.05	-2.41**	-0.45**
	[0.11,0.45]	[-0.11,0.18]	[-0.21,-0.09]	[0.05,0.32]	[-0.04,0.13]	[-2.58,-2.25]	[-0.66,-0.23]
Hypertension	0.41**	0.03	-0.03	0.06	-0.31**	-4.44**	-6.53**
	[0.27,0.56]	[-0.10,0.15]	[-0.08,0.02]	[-0.06,0.18]	[-0.38,-0.24]	[-4.59,-4.29]	[-6.71,-6.35]
Chronic pulmonary disease	-0.25**	-0.02	-0.14**	0.12*	-0.19**	1.42**	4.63**
	[-0.38,-0.12]	[-0.14,0.10]	[-0.19,-0.09]	[0.00,0.23]	[-0.26,-0.13]	[1.29,1.55]	[4.46,4.81]
Rheumatoid arthritis and collagen vascular diseases	0.13	-0.22	-0.26**	0.04	-0.60**	-1.45**	0.17
	[-0.14,0.39]	[-0.48,0.04]	[-0.37,-0.16]	[-0.21,0.29]	[-0.73,-0.47]	[-1.72,-1.18]	[-0.20,0.55]
Coagulation deficiency	1.06**	1.30**	0.48**	0.83**	2.35**	4.76**	7.15**
	[0.83,1.28]	[1.06,1.55]	[0.37,0.58]	[0.60,1.05]	[2.18,2.53]	[4.47,5.05]	[6.80,7.50]
Obesity	0.42**	0.45**	-0.10**	0.55**	0.41**	-1.89**	-3.50**
	[0.24,0.60]	[0.28,0.62]	[-0.17,-0.03]	[0.39,0.71]	[0.31,0.51]	[-2.04,-1.75]	[-3.71,-3.30]
Substance use	0.18	-1.27**	-0.16*	-1.11**	-0.44**	-0.89**	-1.24**
	[-0.11,0.47]	[-1.57,-0.97]	[-0.29,-0.03]	[-1.39,-0.83]	[-0.62,-0.25]	[-1.16,-0.62]	[-1.62,-0.86]
Depression	-0.06	-1.06**	-0.27**	-0.79**	-0.50**	-1.58**	-0.81**
	[-0.25,0.13]	[-1.22,-0.90]	[-0.33,-0.21]	[-0.94,-0.65]	[-0.57,-0.43]	[-1.76,-1.40]	[-1.05,-0.56]

© 2024 Shen Y-C et al. *JAMA Network Open.*

Psychosis	-0.25*	-0.61**	-0.32**	-0.30**	-0.99**	-0.99**	0.17
	[-0.48,-0.02]	[-0.81,-0.42]	[-0.39,-0.24]	[-0.48,-0.11]	[-1.09,-0.89]	[-1.20,-0.78]	[-0.12,0.46]
Hypothyroidism	0.13*	0.04	-0.04	0.08	-0.05	-1.17**	-1.01**
	[0.01,0.25]	[-0.08,0.15]	[-0.09,0.01]	[-0.03,0.19]	[-0.11,0.01]	[-1.30,-1.04]	[-1.18,-0.85]
Paralysis and other neurological disorders	2.09**	9.71**	1.59**	8.13**	3.32**	7.21**	9.89**
	[1.96,2.22]	[9.62,9.81]	[1.54,1.63]	[8.03,8.22]	[3.27,3.38]	[7.10,7.31]	[9.77,10.02]
Chronic peptic ulcer disease	0.53	-0.03	0.10	-0.13	2.83**	-0.80	4.11**
	[-0.11,1.17]	[-0.83,0.76]	[-0.25,0.46]	[-0.86,0.60]	[2.18,3.48]	[-1.64,0.04]	[3.00,5.22]
Weight loss	-0.26*	-0.72**	-0.11*	-0.61**	0.92**	9.95**	20.87**
	[-0.51,-0.00]	[-0.93,-0.51]	[-0.20,-0.03]	[-0.81,-0.40]	[0.77,1.07]	[9.64,10.26]	[20.53,21.21]
Fluid and electrolyte disorders	-0.38**	-0.31**	-0.06*	-0.26**	1.20**	6.05**	10.05**
	[-0.50,-0.26]	[-0.42,-0.20]	[-0.10,-0.01]	[-0.36,-0.15]	[1.14,1.27]	[5.92,6.19]	[9.88,10.22]
Anemia (blood loss and deficiency)	-0.15*	0.94**	0.11**	0.83**	1.23**	0.67**	5.66**
	[-0.30,-0.00]	[0.80,1.08]	[0.05,0.17]	[0.70,0.96]	[1.15,1.32]	[0.52,0.83]	[5.46,5.86]
Post ICD-10 period	1.06**	1.32**	1.16**	0.17	0.08	-5.24**	-10.11**
	[0.64,1.48]	[0.85,1.79]	[0.92,1.40]	[-0.25,0.58]	[-0.14,0.31]	[-5.64,-4.84]	[-10.67,-9.55]
Admitted in 2009	0.00	0.00	0.00	0.00	0.00	0.00	0.00
	[0.00,0.00]	[0.00,0.00]	[0.00,0.00]	[0.00,0.00]	[0.00,0.00]	[0.00,0.00]	[0.00,0.00]
Admitted in 2010	1.53**	0.77**	0.13**	0.64**	0.15**	-0.24	-0.48**
	[1.06,2.01]	[0.59,0.95]	[0.07,0.19]	[0.47,0.81]	[0.07,0.22]	[-0.48,0.01]	[-0.80,-0.16]
Admitted in 2011	5.09**	1.60**	0.60**	1.00**	0.11*	-0.72**	-1.27**
	[4.48,5.71]	[1.40,1.81]	[0.53,0.68]	[0.81,1.19]	[0.02,0.20]	[-0.98,-0.46]	[-1.61,-0.93]
Admitted in 2012	7.39**	2.44**	0.85**	1.59**	0.36**	-0.65**	-2.53**
	[6.68,8.11]	[2.22,2.66]	[0.76,0.93]	[1.39,1.80]	[0.26,0.46]	[-0.92,-0.39]	[-2.88,-2.18]
Admitted in 2013	9.22**	3.57**	1.00**	2.57**	0.49**	-0.92**	-5.44**
	[8.47,9.98]	[3.34,3.80]	[0.91,1.09]	[2.35,2.79]	[0.38,0.59]	[-1.20,-0.65]	[-5.79,-5.08]
Admitted in 2014	7.12**	4.23**	1.14**	3.08**	0.66**	-1.04**	-3.35**
	[6.24,8.00]	[3.99,4.46]	[1.05,1.24]	[2.86,3.31]	[0.55,0.77]	[-1.32,-0.76]	[-3.71,-2.99]

© 2024 Shen Y-C et al. *JAMA Network Open.*

Admitted in 2015	8.52**	4.94**	1.45**	3.48**	1.58**	-1.27**	-3.08**
	[7.62,9.43]	[4.68,5.19]	[1.35,1.56]	[3.24,3.73]	[1.45,1.71]	[-1.56,-0.98]	[-3.47,-2.70]
Admitted in 2016	7.66**	4.71**	0.66**	4.05**	2.14**	2.66**	4.77**
	[6.64,8.68]	[4.19,5.24]	[0.40,0.92]	[3.58,4.52]	[1.88,2.40]	[2.17,3.15]	[4.10,5.44]
Admitted in 2017	8.85**	5.39**	0.65**	4.74**	3.09**	2.36**	4.64**
	[7.84,9.86]	[4.87,5.91]	[0.39,0.91]	[4.27,5.21]	[2.82,3.35]	[1.87,2.85]	[3.98,5.31]
Admitted in 2018	9.69**	5.91**	0.64**	5.28**	4.41**	2.24**	4.11**
	[8.67,10.71]	[5.39,6.44]	[0.38,0.90]	[4.80,5.75]	[4.14,4.68]	[1.74,2.73]	[3.44,4.77]
Admitted in 2019	11.25**	5.77**	0.41**	5.36**	5.11**	1.95**	4.11**
	[10.22,12.28]	[5.24,6.30]	[0.15,0.67]	[4.88,5.84]	[4.84,5.38]	[1.46,2.45]	[3.44,4.78]
constant	70.92**	3.36**	0.26**	3.10**	-0.09	5.34**	11.76**
	[70.31,71.52]	[3.15,3.56]	[0.17,0.34]	[2.91,3.29]	[-0.21,0.02]	[5.10,5.58]	[11.46,12.06]

Note: Linear probability models with community fixed effects. N=1,896,110. *p<0.05, **p<0.01.

© 2024 Shen Y-C et al. *JAMA Network Open.*

eTable 2B. Full Regression Results of the Models Presented in Figure 2 for Communities With No Baseline Access to a Certified Stroke Center

	Admitted to any stroke center	Thrombolytics (overall)	Drip-and-ship	Drip-and-stay	Mechanical thrombectomy	30-day mortality	1-year mortality
on and after year-quarter gaining stroke center within 30-min	38.98**	0.48**	0.15	0.34**	-0.02	-0.28*	-0.50**
	[37.74,40.21]	[0.24,0.73]	[-0.03,0.33]	[0.13,0.54]	[-0.14,0.10]	[-0.56,-0.01]	[-0.84,-0.15]
Black	-1.78**	-1.93**	-0.65**	-1.28**	-0.46**	-3.10**	-1.40**
	[-2.13,-1.42]	[-2.15,-1.72]	[-0.79,-0.52]	[-1.45,-1.11]	[-0.58,-0.35]	[-3.36,-2.83]	[-1.76,-1.04]
Hispanic	-1.02*	-1.00**	-0.30	-0.70**	-0.23	-0.92**	-1.81**
	[-1.85,-0.19]	[-1.55,-0.46]	[-0.62,0.02]	[-1.16,-0.24]	[-0.51,0.05]	[-1.61,-0.24]	[-2.70,-0.93]
Other Race	0.17	-0.79**	-0.35	-0.44	-0.01	-1.63**	-2.44**
	[-0.56,0.90]	[-1.36,-0.22]	[-0.72,0.02]	[-0.91,0.03]	[-0.32,0.31]	[-2.16,-1.09]	[-3.17,-1.71]
Female	-1.55**	-0.40**	-0.13**	-0.27**	0.15**	0.96**	0.21*
	[-1.72,-1.38]	[-0.53,-0.28]	[-0.21,-0.05]	[-0.37,-0.18]	[0.08,0.21]	[0.82,1.11]	[0.03,0.40]
Age 70-74	-0.72**	0.04	0.01	0.02	0.10	0.48**	0.87**
	[-0.99,-0.45]	[-0.17,0.24]	[-0.13,0.15]	[-0.14,0.18]	[-0.01,0.21]	[0.30,0.66]	[0.62,1.12]
Age 75-79	-1.59**	-0.28**	-0.10	-0.19*	-0.07	2.12**	4.73**
	[-1.86,-1.31]	[-0.49,-0.08]	[-0.23,0.04]	[-0.35,-0.03]	[-0.17,0.04]	[1.93,2.31]	[4.47,4.99]
Age 80-84	-3.44**	-1.23**	-0.57**	-0.65**	-0.30**	5.59**	10.92**
	[-3.73,-3.15]	[-1.44,-1.02]	[-0.71,-0.44]	[-0.82,-0.49]	[-0.40,-0.19]	[5.38,5.80]	[10.64,11.20]
Age 85+	-6.35**	-2.43**	-1.16**	-1.27**	-0.97**	14.74**	25.88**
	[-6.66,-6.03]	[-2.62,-2.23]	[-1.29,-1.03]	[-1.42,-1.11]	[-1.07,-0.87]	[14.51,14.96]	[25.60,26.16]
Recurrent stroke	-1.26**	-2.27**	-1.21**	-1.06**	-0.75**	2.06**	5.68**
	[-1.55,-0.97]	[-2.48,-2.07]	[-1.34,-1.08]	[-1.23,-0.90]	[-0.86,-0.65]	[1.80,2.33]	[5.34,6.01]
Peripheral vascular disease	1.20**	-0.93**	-0.50**	-0.42**	0.29**	0.33**	1.95**
	[0.88,1.51]	[-1.12,-0.73]	[-0.63,-0.37]	[-0.58,-0.27]	[0.18,0.40]	[0.09,0.57]	[1.64,2.25]

© 2024 Shen Y-C et al. *JAMA Network Open.*

Pulmonary circulation disorders	2.40**	0.83**	0.24*	0.59**	0.77**	4.53**	7.65**
	[1.91,2.90]	[0.49,1.17]	[0.03,0.46]	[0.31,0.87]	[0.58,0.96]	[4.07,5.00]	[7.10,8.20]
Diabetes	-1.04**	-1.80**	-0.74**	-1.06**	-0.61**	0.16*	2.03**
	[-1.22,-0.86]	[-1.93,-1.67]	[-0.82,-0.65]	[-1.16,-0.96]	[-0.68,-0.55]	[0.01,0.30]	[1.84,2.22]
Kidney failure	-1.32**	-1.89**	-1.17**	-0.72**	-0.85**	2.63**	8.17**
	[-1.55,-1.08]	[-2.04,-1.74]	[-1.27,-1.07]	[-0.84,-0.60]	[-0.93,-0.77]	[2.44,2.83]	[7.91,8.42]
Liver disease	-0.30	-3.00**	-1.53**	-1.47**	-0.52**	3.08**	8.16**
	[-1.14,0.54]	[-3.56,-2.44]	[-1.87,-1.18]	[-1.93,-1.01]	[-0.87,-0.17]	[2.34,3.83]	[7.22,9.11]
Cancer	0.32	-3.40**	-1.68**	-1.72**	-0.38**	14.29**	30.53**
	[-0.09,0.74]	[-3.68,-3.13]	[-1.85,-1.51]	[-1.95,-1.50]	[-0.55,-0.22]	[13.82,14.76]	[30.01,31.06]
Dementia	-3.60**	-3.70**	-1.83**	-1.87**	-1.73**	3.73**	11.73**
	[-3.89,-3.30]	[-3.90,-3.51]	[-1.95,-1.71]	[-2.03,-1.71]	[-1.82,-1.63]	[3.45,4.01]	[11.39,12.08]
Valvular disease	1.25**	0.05	-0.05	0.11	0.01	-2.60**	-0.87**
	[0.91,1.58]	[-0.17,0.27]	[-0.19,0.09]	[-0.07,0.28]	[-0.10,0.13]	[-2.84,-2.36]	[-1.19,-0.56]
Hypertension	1.60**	0.30**	0.21**	0.08	-0.13**	-4.66**	-6.53**
	[1.35,1.84]	[0.13,0.46]	[0.11,0.32]	[-0.05,0.21]	[-0.22,-0.05]	[-4.87,-4.45]	[-6.77,-6.28]
Chronic pulmonary disease	-1.70**	-0.41**	-0.28**	-0.14*	-0.25**	1.76**	5.35**
	[-1.92,-1.47]	[-0.57,-0.26]	[-0.38,-0.17]	[-0.26,-0.01]	[-0.33,-0.17]	[1.57,1.94]	[5.12,5.59]
Rheumatoid arthritis and collagen vascular diseases	-0.35	-0.94**	-0.55**	-0.38**	-0.40**	-1.72**	-0.06
	[-0.82,0.12]	[-1.28,-0.59]	[-0.77,-0.34]	[-0.66,-0.10]	[-0.57,-0.23]	[-2.12,-1.33]	[-0.59,0.47]
Coagulation deficiency	4.01**	1.28**	0.55**	0.73**	1.96**	5.37**	7.14**
	[3.56,4.47]	[0.93,1.63]	[0.32,0.78]	[0.45,1.01]	[1.73,2.20]	[4.92,5.82]	[6.62,7.66]
Obesity	1.05**	0.64**	0.08	0.56**	0.43**	-1.72**	-3.26**
	[0.72,1.38]	[0.41,0.87]	[-0.08,0.24]	[0.38,0.74]	[0.29,0.56]	[-1.93,-1.51]	[-3.54,-2.97]
Substance use	-0.70*	-1.88**	-0.74**	-1.14**	-0.42**	-0.32	-0.24
	[-1.25,-0.15]	[-2.31,-1.46]	[-1.02,-0.45]	[-1.47,-0.81]	[-0.67,-0.17]	[-0.73,0.10]	[-0.80,0.32]
Depression	-0.65**	-1.01**	-0.48**	-0.53**	-0.42**	-1.91**	-1.19**
	[-0.99,-0.31]	[-1.22,-0.80]	[-0.61,-0.35]	[-0.70,-0.36]	[-0.51,-0.34]	[-2.17,-1.65]	[-1.54,-0.85]

© 2024 Shen Y-C et al. *JAMA Network Open.*

Psychosis	-0.59**	-0.28	-0.35**	0.08	-0.79**	-0.86**	0.14
	[-1.00,-0.18]	[-0.56,0.00]	[-0.53,-0.17]	[-0.15,0.30]	[-0.92,-0.66]	[-1.18,-0.55]	[-0.29,0.56]
Hypothyroidism	0.29**	-0.08	-0.05	-0.04	-0.12**	-1.47**	-1.50**
	[0.07,0.50]	[-0.24,0.07]	[-0.15,0.05]	[-0.16,0.09]	[-0.20,-0.04]	[-1.65,-1.29]	[-1.73,-1.26]
Paralysis and other neurological disorders	5.54**	8.59**	3.40**	5.19**	2.68**	7.04**	9.36**
	[5.31,5.77]	[8.47,8.72]	[3.31,3.50]	[5.08,5.30]	[2.61,2.74]	[6.90,7.19]	[9.18,9.54]
Chronic peptic ulcer disease	1.77**	-0.69	-0.43	-0.26	2.01**	0.82	4.99**
	[0.52,3.01]	[-1.80,0.42]	[-1.18,0.32]	[-1.11,0.60]	[1.19,2.83]	[-0.42,2.06]	[3.39,6.59]
Weight loss	1.22**	-0.63**	-0.37**	-0.26*	0.62**	10.28**	20.34**
	[0.69,1.75]	[-0.93,-0.33]	[-0.56,-0.18]	[-0.49,-0.03]	[0.44,0.81]	[9.83,10.73]	[19.84,20.84]
Fluid and electrolyte disorders	-0.19	-0.50**	-0.28**	-0.22**	1.11**	6.22**	9.92**
	[-0.40,0.03]	[-0.64,-0.35]	[-0.37,-0.18]	[-0.34,-0.11]	[1.03,1.20]	[6.03,6.40]	[9.69,10.14]
Anemia (blood loss and deficiency)	-0.24	0.41**	0.00	0.41**	0.89**	0.65**	5.59**
	[-0.51,0.03]	[0.22,0.60]	[-0.12,0.12]	[0.26,0.56]	[0.78,1.01]	[0.42,0.89]	[5.29,5.89]
Post ICD-10 period	3.19**	1.21**	1.78**	-0.57**	-0.08	-4.80**	-9.25**
	[2.45,3.93]	[0.61,1.81]	[1.33,2.23]	[-1.00,-0.14]	[-0.34,0.17]	[-5.36,-4.24]	[-9.99,-8.50]
Admitted in 2009	0.00	0.00	0.00	0.00	0.00	0.00	0.00
	[0.00,0.00]	[0.00,0.00]	[0.00,0.00]	[0.00,0.00]	[0.00,0.00]	[0.00,0.00]	[0.00,0.00]
Admitted in 2010	3.58**	0.85**	0.22**	0.63**	0.12**	-0.43*	-0.58*
	[3.05,4.10]	[0.65,1.06]	[0.11,0.33]	[0.45,0.81]	[0.04,0.21]	[-0.79,-0.08]	[-1.02,-0.14]
Admitted in 2011	9.14**	2.14**	1.34**	0.80**	0.09*	-0.61**	-0.74**
	[8.52,9.76]	[1.92,2.36]	[1.20,1.48]	[0.62,0.98]	[0.01,0.18]	[-0.97,-0.26]	[-1.18,-0.31]
Admitted in 2012	13.52**	2.96**	1.73**	1.23**	0.29**	-0.81**	-2.35**
	[12.81,14.23]	[2.72,3.20]	[1.58,1.89]	[1.04,1.42]	[0.19,0.38]	[-1.16,-0.46]	[-2.80,-1.91]
Admitted in 2013	18.07**	4.10**	2.29**	1.81**	0.39**	-0.82**	-4.91**
	[17.31,18.82]	[3.85,4.36]	[2.12,2.45]	[1.61,2.02]	[0.28,0.49]	[-1.18,-0.46]	[-5.36,-4.46]
Admitted in 2014	21.45**	5.07**	2.69**	2.38**	0.59**	-0.90**	-2.86**
	[20.65,22.25]	[4.80,5.33]	[2.51,2.86]	[2.16,2.60]	[0.49,0.70]	[-1.26,-0.54]	[-3.31,-2.40]

© 2024 Shen Y-C et al. *JAMA Network Open.*

Admitted in 2015	24.56**	6.04**	3.37**	2.67**	1.29**	-1.19**	-2.67**
	[23.71,25.40]	[5.74,6.34]	[3.17,3.57]	[2.43,2.91]	[1.16,1.43]	[-1.57,-0.81]	[-3.15,-2.19]
Admitted in 2016	22.96**	5.65**	2.11**	3.54**	1.83**	2.28**	4.78**
	[21.85,24.07]	[5.00,6.31]	[1.63,2.60]	[3.06,4.02]	[1.54,2.12]	[1.62,2.95]	[3.91,5.65]
Admitted in 2017	25.43**	6.23**	2.33**	3.90**	2.80**	2.13**	4.73**
	[24.31,26.56]	[5.57,6.89]	[1.84,2.82]	[3.41,4.39]	[2.50,3.09]	[1.46,2.80]	[3.85,5.61]
Admitted in 2018	28.19**	6.94**	2.56**	4.38**	4.05**	1.91**	4.08**
	[27.07,29.32]	[6.28,7.61]	[2.07,3.05]	[3.89,4.87]	[3.74,4.35]	[1.24,2.57]	[3.20,4.96]
Admitted in 2019	30.82**	7.29**	2.57**	4.72**	4.85**	1.67**	4.10**
	[29.67,31.97]	[6.62,7.95]	[2.07,3.06]	[4.22,5.22]	[4.54,5.16]	[1.01,2.34]	[3.22,4.99]
constant	25.52**	2.39**	0.60**	1.79**	-0.19**	6.29**	12.48**
	[24.89,26.14]	[2.12,2.66]	[0.42,0.77]	[1.58,2.01]	[-0.33,-0.06]	[5.94,6.63]	[12.06,12.91]

Note: Linear probability models with community fixed effects. N= 957,176. *p<0.05, **p<0.01.

© 2024 Shen Y-C et al. *JAMA Network Open.*

eTable 3. Risk-Adjusted Percentage Point Changes in Treatment and Mortality Outcomes When a Newly Certified Stroke Center Becomes Available Within a 30-Minute Drive, Stratified by Baseline Stroke Center Access and Highest Certification Level

	Thrombolytic (overall)	Drip-and-ship	Drip-and-stay	Mechanical thrombectomy	30-day mortality	1-year mortality
Community had pre-existing stroke center access at baseline (N=1,896,110)						
on and after year-quarter gaining certified stroke center within 30-min						
ASRH	0.03	0.83**	-0.80	-0.07	-0.10	0.28
	[-1.00,1.06]	[0.24,1.43]	[-1.67,0.06]	[-0.61,0.47]	[-1.08,0.88]	[-0.90,1.47]
PSC	0.03	0.10*	-0.07	-0.15**	0.02	0.03
	[-0.16,0.22]	[0.02,0.18]	[-0.25,0.11]	[-0.25,-0.06]	[-0.19,0.22]	[-0.24,0.29]
TSC or CSC	0.27*	-0.20**	0.47**	0.34**	-0.01	-0.49**
	[0.05,0.50]	[-0.29,-0.11]	[0.26,0.69]	[0.23,0.45]	[-0.24,0.22]	[-0.79,-0.19]
Community had no stroke access at baseline (N=957,176)						
on and after year-quarter gaining certified stroke center within 30-min						
ASRH	0.41	1.58**	-1.17**	0.19	-0.12	-0.36
	[-0.07,0.89]	[1.16,2.01]	[-1.50,-0.84]	[-0.06,0.45]	[-0.62,0.37]	[-0.95,0.23]
PSC	0.43**	-0.28**	0.71**	-0.13	-0.36*	-0.55**
	[0.16,0.70]	[-0.46,-0.10]	[0.47,0.95]	[-0.27,0.00]	[-0.66,-0.05]	[-0.94,-0.16]
TSC or CSC	2.26**	-1.18**	3.44**	0.82**	0.14	-0.39
	[1.59,2.93]	[-1.57,-0.79]	[2.79,4.09]	[0.41,1.23]	[-0.49,0.78]	[-1.21,0.43]

Note: Community-fixed effects models are adjusted for patient demographics (age, sex, race, ethnicity) and comorbid conditions, and controlled for yearly trend. ASRH indicates Acute Stroke Ready Hospital. ASRH indicates Acute Stroke Ready Hospital. PSC indicates Primary Stroke Center. TSC indicates Thrombectomy Capable Stroke Center. CSC indicates Comprehensive Stroke Center. * p<0.05 ** p<0.01.

© 2024 Shen Y-C et al. *JAMA Network Open.*
