# Supplementary material for: Treatments and Patient Outcomes Following Stroke Center Expansion
Source: JAMA Netw Open. 2024 Nov 13;7(11):e2444683. doi: 10.1001/jamanetworkopen.2024.44683 (PMC11561690; doi:10.1001/jamanetworkopen.2024.44683)
Supplement: Supplement 2. — Data Sharing Statement [file jamanetwopen-e2444683-s002.pdf]

Data Sharing Statement

Shen. Treatments and Patient Outcomes Following Stroke Center Expansion. *JAMA Netw Open*. Published November 13, 2024. doi:10.1001/jamanetworkopen.2024.44683

Data
Data available: No
